# Bridging the Gap: commodifying infrastructure spatial dynamics with crowdsourced smartphone data

**DOI:** 10.1038/s44172-024-00243-y

**Published:** 2024-07-04

**Authors:** Liam Cronin, Soheil Sadeghi Eshkevari, Thomas J. Matarazzo, Sebastiano Milardo, Iman Dabbaghchian, Paolo Santi, Shamim N. Pakzad, Carlo Ratti

**Affiliations:** 1 Lehigh University, Civil & Environmental Engineering Department, Bethlehem, USA; 2 MIT, Senseable City Laboratory, Cambridge, USA; 3 United States Military Academy, Department of Civil & Mechanical Engineering, West Point, USA

**Keywords:** Civil engineering, Applied physics, Mechanical engineering

## Abstract

Structural information deficits about aging bridges have led to several avoidable catastrophes in recent years. Data-driven methods for bridge vibration monitoring enable frequent, accurate structural assessments; however, the high costs of widespread deployments of these systems make important condition information a luxury for bridge owners. Smartphone-based monitoring is inexpensive and has produced structural information, i.e., modal frequencies, in crowdsensing applications. Even so, current methods cannot extract spatial vibration characteristics with uncontrolled datasets that are needed for damage identification. Here we present an extensive real-world study with crowdsourced smartphone-vehicle trips within motor vehicles in which we estimate absolute value mode shapes and simulate damage detection capabilities. Our method analyzes over 800 trips across four road bridges with main spans ranging from 30 to 1300 m in length, representing about one-quarter of bridges in the United States. We demonstrate a bridge health monitoring platform compatible with ride-sourcing data streams that check conditions daily. The result has the potential to commodify data-driven structural assessments globally.

## Abstract (web summary)

Liam Cronin and colleagues develop an end-to-end approach for crowdsourcing vibration data from cell phones in vehicles to extract bridge vibration characteristics known to be sensitive to structural damage.

## Introduction

Ubiquitous smartphones have normalized the distributed collection of data in everyday life. Sensor arrays in modern smartphones enable unprecedented measurements of an individual’s activities. In contrast to increasing digital capabilities, modern society faces significant infrastructure deficits. Knowledge gaps regarding the conditions of infrastructure systems have created vulnerabilities to sudden and unexpected losses in service and have ultimately produced an infrastructure-funding gap. For instance, at current investment levels, it would take about 50 years for the U.S. Department of Transportation (DOTs) to resolve outstanding repairs. This projection is conservative as it does not include any new bridge maintenance issues that would arise. The 10-year infrastructure-funding gap for roadways and transit is over one trillion USD^1^. Furthermore, the exposure of infrastructure to natural disasters amplifies uncertainties in asset management. In regions with high risks of multiple natural hazards, e.g., earthquakes, coastal flooding, cyclones, etc., transportation infrastructure is subject to significant losses. For example, road bridges in China account for about 29% of the expected annual damage caused by natural hazards^2^. Bridges and other road infrastructure are vulnerable to the rising rates of natural disasters and extreme events in response to climate change and rapid growth in the global human footprint^3–5^. In particular, flooding and other hydraulic events are a leading cause of bridge failures in the U.S.^6^, the U.K.^7^, India^8^, and other countries. This emphasizes the importance of incorporating accurate models of bridges and their exposure to extreme events in life-cycle analyses^9^.

Modern structural health monitoring (SHM) techniques are highly capable of determining critical physical characteristics of bridges based on sensor data. SHM encompasses a wide range of services such as modal identification, damage identification and localization, digital twin modeling, risk quantification, and disaster response^10,11^. Advances in sensing and actuation have led to applications utilizing computer vision and robotics, e.g., automatic crack detection, drone-based inspections, etc., which are designed to improve the retrieval of structural condition information^12–14^. However, the costs associated with implementing these techniques (even in their simplest forms) have proven unattainable for most bridge owners. Sensing technology is not part of routine bridge inspections: U.S. National Bridge Inspection Standards only require that each bridge is inspected visually in 24-month intervals. A widespread need for monitoring approaches that are accurate, easy to implement, and cost-effective has helped spark interest in low-cost alternatives, such as the use of mobile sensor networks^15^ and smartphones^16^ in SHM.

In the last two decades, researchers^15,17–23^ have established the advantages that mobile sensor networks have over traditional “stationary” ones in measuring bridge vibrations. Two key findings for mobile sensor networks are (i) few devices are needed to determine structural dynamical properties, which enables widespread bridge monitoring at a lower cost; and (ii) mobile sensors efficiently capture dense spatial vibration information, e.g., high-resolution structural mode shapes^24–28^. Simultaneously, recent research utilizing smartphones for structural health monitoring have considered various contexts, primarily focusing on stationary (not mobile) applications. Stationary smartphones have accurately measured acceleration signals on civil structures, and they have been used for the identification of modal properties of pedestrian and highway bridges^16,29–31^.

There have been very few studies that have considered smartphone data collected in moving vehicles, and even fewer within the specific context of crowdsourced smartphone data collected within moving automobiles. Simulations of crowdsourced smartphone data have been generated and analyzed to identify bridge modal frequencies^22^. In addition, vehicles instrumented with multiple accelerometers, smartphones, and positioning systems were used to detect road abnormalities, including bridge expansion joints, but did not estimate any bridge dynamical properties^32^. In one study, researchers collected 42 smartphone datasets in motor vehicle trips over the Harvard Bridge and extracted indicators of three bridge modal frequencies^33^. In another study, researchers developed a method to extract spatial information, e.g., absolute value mode shapes, from an asynchronous network of mobile sensors and validated this technique using a network of moving smartphones on a beam in a lab setting and simulated the effects of vehicle-bridge interaction^34^. A pilot study focusing on micromobility used data collected by smartphones in bicycle trips over a footbridge and identified its first absolute mode shape^35^—the study collected about 40 datasets, primarily biking trips with speeds below 8 kph.

The most relevant recent work presented multiple real-world applications in which about 450 crowdsourced smartphone-vehicle-trip (SVT) datasets were used to accurately determine the modal frequencies of two real-world highway bridges^36^. Notably, this study used a variety of crowdsourced data from several external sources, such as a ridesourcing company and a bridge maintenance crew. These results emphasized the unique and significant advantages of crowdsourced data collected within moving automobiles and in different controllability environments. “Pre-existing” mobile sensor networks^37^ such as ridesharing data, municipal transit data, etc., provide an unprecedented potential for high velocity and large-scale data streams. Modal frequency identification is an essential first step in monitoring the dynamics and condition of a bridge. While modal frequencies have been successfully used to identify structural damage in real-world applications, certain modal frequencies, e.g., lower modes, can be insensitive to damage and simultaneously sensitive to normal environmental changes^38–45^, which can reduce their effectiveness as a damage-sensitive feature.

Spatial vibration information, such as mode shapes, are effective damage-sensitive features and can be captured efficiently by mobile sensors^27,34,35^. Structural mode shapes and their curvatures are sensitive to local and global damage while less sensitive to environmental variations^46^. Over time, as structural damage develops, the deviations in mode-shape-based metrics^47–50^ can lead to the identification of both the presence and location of the damage. Similarly, signal processing techniques, e.g., wavelet transforms, have been used to identify mode shape discontinuities and attributed them to local damage^51–56^. For these reasons, mode shapes and mode shape curvatures are widely studied and used for damage detection, and localization^57–61^. The development of damage detection methods is an open research topic. A robust and accurate monitoring system must incorporate several layers of information into a condition report, such as available environmental data, dynamical properties, and damage-sensitive features to counteract the detrimental effects of sensor noise, modal property estimation inaccuracies, and environmental factors.

**Fig. 1:**
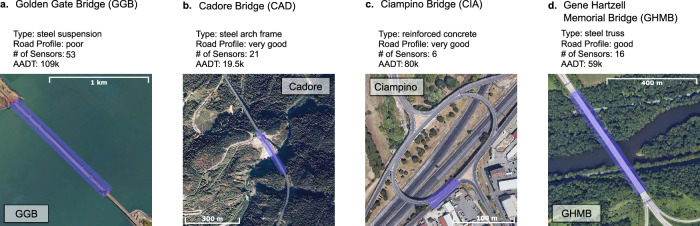
Aerial views and general information for the four case studies (source: Google Earth), and the monitored sections are highlighted in the photos. **a** Golden Gate Bridge, **b** Cadore Bridge, **c** Ciampino Bridge, and **d** Gene Hartzell Memorial Bridge. These bridges are monitored with traditional fixed sensor networks, and results are used as a baseline to compare with the mobile sensing campaign. In summary, these bridges display a wide range of characteristics with span-lengths varying from 56 to 1280 m and consist of four distinct structural systems.

This paper proposes a method for identifying absolute value mode shapes (AMS) of highway bridges using highly uncontrolled mobile sensing data: crowdsourced SVT data. The method is validated throughout five real-world applications on four distinct bridges, Fig. 1: The Golden Gate Bridge (USA), the Cadore Bridge (Italy), the Ciampino Bridge (Italy), and the Gene Hartzell Memorial Bridge (USA). In total, 884 SVT datasets were collected across four bridges, and in three types of controllability environments. These bridges have distinctly different locations, designs, and traffic volumes. Notably, the lengths of the largest span vary from 30 m to 1.3 km, a range that represents 32% of US bridges.

The proposed method has three key benefits: (1) it is entirely based on SVT data which can be sourced from common smartphones and does not require supplementary sensory or GIS information, (2) with ever-expanding smartphone penetration rates, it can streamline up-to-date AMS estimations regularly, which is critical for long-term, reference-based health monitoring, and (3) the spatial resolution of the AMS is high, which enables immediate applications to broader SHM services such as damage identification. These features can be configured with a software-as-service (SaS) system for automated data collection, preprocessing, and cloud-based storage that can provide tools for near-real-time evaluations. As a whole, these capabilities demonstrate a platform that reforms the costly, labor-intensive task of acquiring bridge condition monitoring data, such that “luxury” information is made into an affordable, readily available good—a commodity—for bridge owners.

## Method

**Fig. 2:**
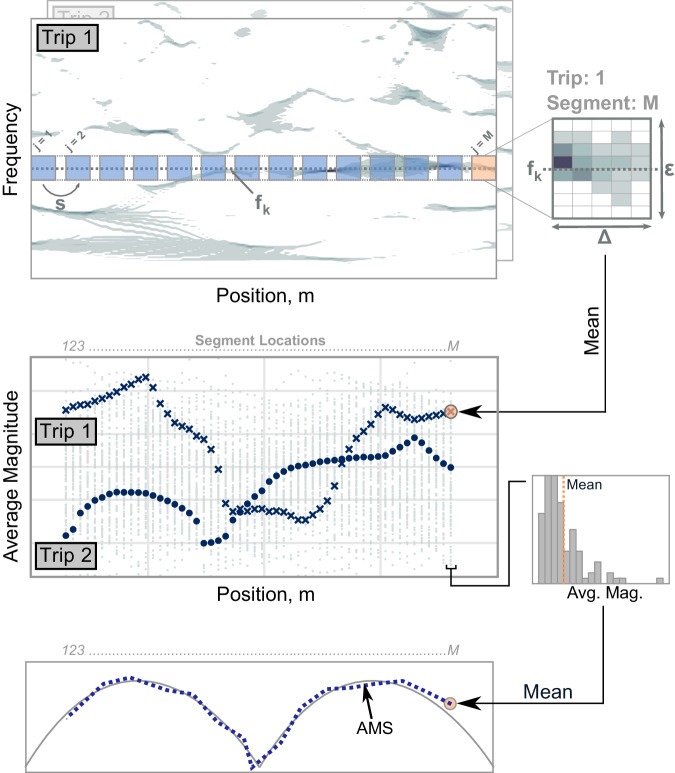
Overview of the methodology: the synchrosqueezed wavelet transform converts each acceleration time series to the time-frequency domain, and the instantaneous magnitudes are calculated. The bridge is divided into overlapping segments; the width (Δ) and spatial stride (*S*) are two parameters of the method. Averaging the magnitudes in each segment for each trip at the modal frequencies yields a distribution of magnitudes at each location, the mean of which is the AMS estimate. Including a small bandwidth (*ϵ*) around the modal frequency in the averaging process leads to robust results on noisy datasets. Detailed information is given in “Methods”.

The process for identifying AMS is represented graphically in Fig. 2. The “Methods” section gives a detailed explanation of the proposed methodology for AMS identification. After basic preprocessing steps, the synchrosqueezed wavelet transform is applied to the SVT acceleration signals. To initialize the aggregation process, the bridge is segmented, defined by the width of the segments, Δ, and the spacing between segment centers *S*. In addition, narrow frequency bands, with bandwidth *ϵ*, are defined based on prior knowledge of modal frequencies. It is key to emphasize that this method uses prior estimates of natural frequencies. An approach for determining modal frequencies from SVT data is reviewed in the SI along with validations on the presented case studies^36^.

The AMS is estimated by averaging the magnitude of each bridge segment around a natural frequency. The dimensions of the matrix depicted having width Δ and height *ϵ*, being averaged, are contingent on the number of samples taken within the bridge segment and discretization in frequency of the wavelet transform. This would be written as a conditional index, selecting measurements over a bridge segment and within a bandwidth. In the middle panel of Fig. 2, the mean magnitude for all trips and segments are plotted at the central frequency as a function of segment centers. The mean value over all trips at each location is extracted as the AMS for the modal frequency. Varying the segment width, segment center spacing, and bandwidth, the spatial resolution of the AMS can be adjusted.

The accuracy of the AMS is evaluated using modal assurance criteria (MAC), which produces a value between 0 and 1 that measures the quality (high quality generally being above 0.90) of an estimated mode shape by comparing it with the reference mode shape^62^. The analysis to find the reference mode shape for all cases is presented in Supplementary Note 2. This includes data collection campaigns completed by the authors and references to prior works.

## Results

This study examines the efficacy of the proposed method on four real-world bridges with distinct locations, designs, and traffic volumes. The applications considered a broad set of SVT conditions; data were collected using different vehicles, e.g., sedans, minivans, etc., in three types of environments, controlled, partially controlled, and uncontrolled, by a variety of smartphone models, and with sample sizes ranging from 50 to 200. Details of the SVT data in each application are provided in the following sections; however, detailed information on data collection, processing, and complete dataset descriptions are found in the “Methods” and Supplementary Note 1. Consistent with prior work using mobile sensor networks, the resulting AMS has a very high spatial resolution; the applications below produce mode shapes with 50 points. In traditional SHM applications with fixed sensors, the spatial resolution is limited by the number of simultaneous sensors. A mode shape with 50 points requires a network of 50 synchronized sensors. All applications produced highly accurate AMS as measured by MAC values of 0.94 and above. These results provide strong evidence that supports the collection and analyses of crowdsourced SVT data for determining spatial vibration characteristics of existing bridges.

### Golden Gate Bridge

The landmark Golden Gate Bridge (GGB) connects San Francisco to Marin County with an annual average daily traffic (AADT) of 109,000. The suspension bridge has a main span of 1280 m over San Francisco Bay. Due to its structural flexibility, the modal frequencies are much lower than the other bridges in this study. The first three vertical modal frequencies are 0.106, 0.132, and 0.169 Hz^63^, and the fundamental transverse mode has been estimated within 0.05, and 0.095^64,65^ Hz. For SVT data, two distinct datasets are evaluated. The first dataset, GGB-C, consists of 102 trips collected in a controlled environment^36^, i.e., key variables such as vehicle velocity and smartphone orientation were controlled. Uber provided the second, limited SVT dataset GGB-UC from its ride-hailing fleet (with no possibility for a higher volume of data sharing), which consists of 50 trips made by a diverse set of drivers and vehicles for an experimental verification with uncontrolled data.

**Fig. 3:**
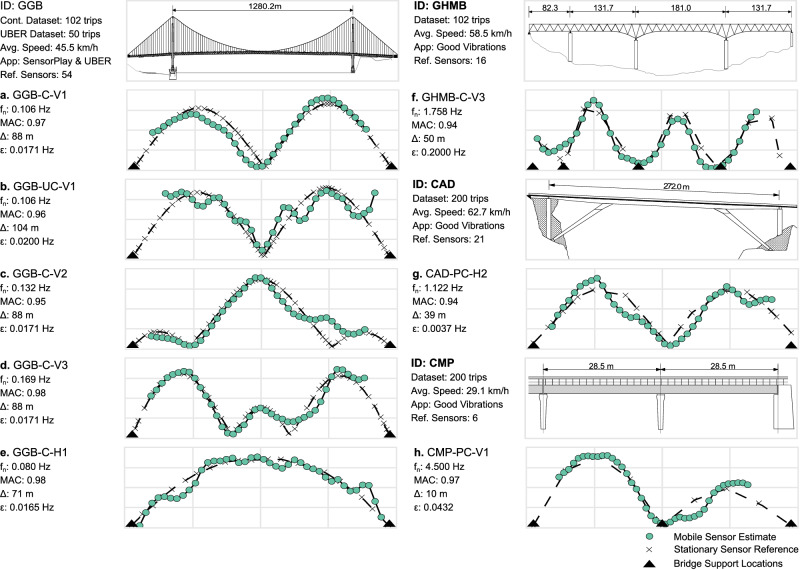
AMS estimates for all case studies. **a**–**e** Golden Gate Bridge, **f** Gene Hartzell Memorial Bridge, **g** Cadore Bridge, and **h** Ciampino Bridge. The estimate from the mobile sensing data is shown with green markers and a solid black line. While the reference is displayed with a dotted line and black X markers. Each X represents the location of the fixed sensor on the bridge during the original study. Last, black triangles show structural supports in the direction of the mode.

In summary, four modes were identified from the controlled data (GGB-C): three vertical and one transverse. In addition, the first vertical mode was identified from the uncontrolled data (GGB-UC). MAC values were calculated by comparing mode shapes at the locations of the fixed sensors from prior work^63^ (42 locations for vertical modes and 19 for the transverse mode). The MAC values for all AMS estimates for both SVT datasets are 0.95 or above, as noted in Fig. 3a–e. Furthermore, a transverse mode was identified using SVT data, and the corresponding AMS was found with a MAC value of 0.98. Lastly, it is worth noting that the AMS of the first vertical mode was identified reasonably well from the uncontrolled data, as indicated with a MAC value of 0.96 and shown in Fig. 3b.

### Gene Hartzell Memorial Bridge

The Gene Hartzell Memorial Bridge (GHMB) spans the Lehigh river along route 33 in Easton, PA, United States with an AADT of 59,000. The bridge structure is a steel truss with four spans and a total length of 540 m with a main span of 180 m. The first three vertical modes are found to be 0.87, 1.34, and 1.78 Hz (see Supplementary Note 2). The SVT data called GHMB is a controlled dataset of 332 trips collected by the research team’s vehicles and the Good Vibrations App (more information in “Methods” section). For data collection, the team drove at a set of prescribed speeds detailed in Supplementary Note 1. The mean speed across all trips was 82.25 km/h, which is slightly below the speed limit of 104.6 km/h.

For analysis, the dataset was subdivided to consider the slowest 102 trips. The idea of dividing large datasets into potentially “more informative” subsets is considered further in the following section. With this subset, the AMS for the third vertical was identified (Fig. 3f). The corresponding MAC value was 0.94, which was calculated using 14 reference locations. The first two vertical modes were not reliably identified, emphasizing that some vibration modes may not be observable in specific SVT subsets.

### Cadore Bridge

The Cadore Bridge is a 272 m long, steel-rigid-arch structure in northern Italy with an AADT of 20,000. The bridge was inspected and rehabilitated in 2011, and the fundamental natural frequencies are reported as 0.68, 1.24, and 1.80 Hz for the first three vertical modes. In addition, the bridge was inspected again with a fixed sensor network in 2021, and vertical and transverse model properties were extracted (see Supplementary Note 2).

The SVT data called CAD-PC consists of 884 partially-controlled samples recorded using the Good Vibrations app by ANAS S.p.A. operators. The vehicle speeds are, on average, 62.7 km/h. Of the full dataset, the 200 slowest trips were selected for further analysis. With this subset of data, Fig. 3g displays the second transverse mode with a frequency of 1.122 Hz. The MAC value between the estimated AMS and the reference shape (17 locations) was 0.94.

### Ciampino Bridge

This bridge is one segment of an elevated intersection located in the Ciampino district of Rome, Italy. The AADT for this bridge is ~82,000. The segment consists of two adjacent continuous spans of reinforced concrete with a total length of 56.7 m. Since September 2020, the bridge has been monitored using a fixed sensor network with the first two natural frequencies identified as 4.5 and 6.8 Hz (See Supplementary Note 2). The SVT data called CMP-PC consists of 992 partially-controlled datasets collected by ANAS S.p.A. using the Good Vibrations App. The results shown in Fig. 3h are from the aggregation of the 200 samples with the lowest speeds. The identified AMS in Ciampino Bridge has a MAC of 0.97 when compared to the reference at six locations.

### Study of data quantity and quality

Many factors influence how the bridge vibrations are transmitted from the road surface to the vehicle cabin and thereby affect the ability to extract accurate dynamics information from the recorded signal. Vehicle speed and road surface roughness are known to be highly impactful^66^. While the available data is insufficient in size and variety for a broad assessment of trends and sensitivities with respect to individual trips or groups of trips, e.g., subsets based on metadata, there are useful observations to note on the apparent effects of dataset size and vehicle speed.

**Fig. 4:**
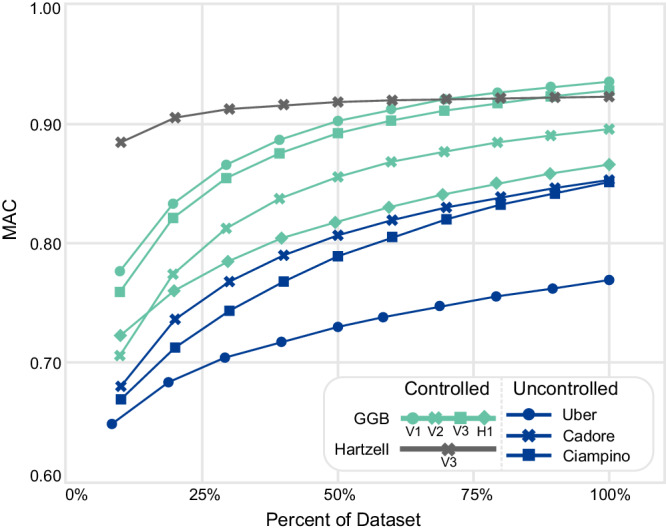
MAC of each case study while varying the dataset size. This is calculated by bootstrapping a significantly large number of sample sets such that the mean MAC converged: 100,000 datasets.

In Fig. 4, the relationship between sample size and MAC value is presented for the identified AMSs. The sample size is presented in terms of the percentage of the entire sample set. For each percentage level, subsets were randomly selected with replacement from the full dataset (i.e., sample bootstrapping), and for each subset, the MAC value was calculated. This process was repeated a sufficiently large number of times so the average MAC across all subsets converged (100,000 sample sets). From Fig. 4, in all cases, without exception, the accuracy of identified AMSs monotonically increased with sample size. Furthermore, since these curves are still increasing, we would likely still see improvement in accuracy with additional data, potentially improving results with lower MAC values. The extent of this improvement varied with each mode and with respect to the controllability of the SVT data. In one case, increasing sample sizes rose the MAC value of the first mode from Uber data (*N* = 50) by 31%. There are two further observations: (1) the rate at which accuracy increases decreases with sample size, and (2) accuracy increases at a slower rate for the uncontrolled data. This suggests that when compared to uncontrolled trips, fewer controlled trips would be needed to achieve a certain AMS accuracy level for a given mode. For instance, consider the first vertical mode of the GGB: the MAC value from 50% of the controlled data (50 samples used) is about 0.90, which is considerably higher than 0.75, the value from the Uber data (50 samples used).

**Fig. 5:**
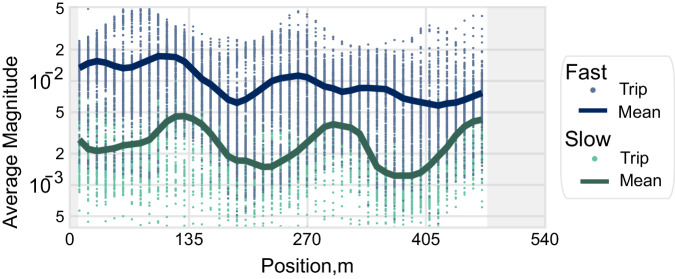
The GHMB dataset is filtered by speed; the 231 fastest speeds are shown in blue, and the 102 slowest speeds are in green. The solid lines are the means for each speed subset.

In a crowdsourced data collection campaign where results from a large number of samples are averaged, it is possible to achieve accurate results even if a few individual trips have excessive noise. In other words, while all datasets may be weighted equally during aggregation, it is expected that may not equally contribute to structural dynamics information. Figure 5 highlights the band-passed frequency-space plot (mode V3 in GHMB-C) for two speed subsets, where blue points represent faster trips and green slower trips, respectively. Overall, a stark difference is visible in Fig. 5 where trips with higher vehicle speeds consistently possess larger magnitudes. Based on prior work, we hypothesize that the increased magnitudes predominantly increase signal noise and a lower signal-to-noise (SNR) ratio, as there is a reduced data duration and the vehicle dynamical response to the road irregularities is intensified^15,66^.

Considerations on apparent dataset “quality” becomes especially important when data volumes evolve to larger orders of magnitude, i.e., tens of thousands of datasets. For instance, crowdsensing platforms with monthly data streams on the order of millions would greatly benefit from preprocessing tools that can filter out datasets that may be noisy or less likely to positively impact SHM features of interest. This process could operate by flagging datasets based on metadata analyses, e.g., speed, smartphone, vehicle type, etc., that strongly correlated with less accurate AMS.

### Scalable bridge network monitoring system

Data-driven bridge health monitoring using sensors sensor networks is a well-established field of scientific research with successful damage detection case studies dating back to the late 1990s. Yet, bridge owners rarely leverage this technology, such that the vast majority of bridge management decisions are made without bridge dynamics information. An appealing vibration monitoring system must be cost-effective and provide a clear advancement in condition information retrieval.

**Fig. 6:**
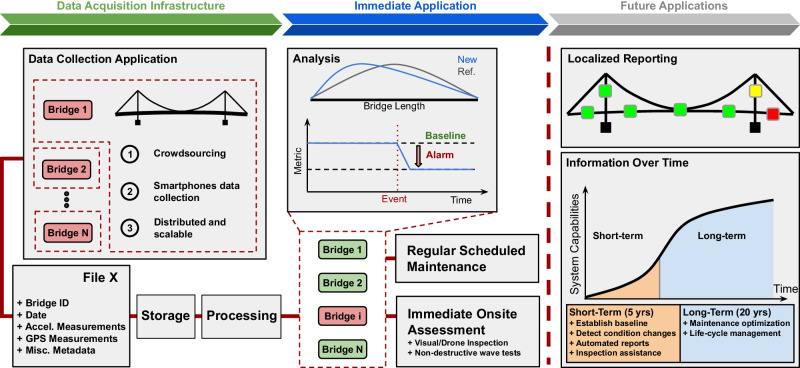
Flow chart of the scalable system identification platform. This paper demonstrates the first two panels: automated data collection with the Good Vibrations app, system identification on four bridges, and a damage detection simulation. Future applications will leverage larger datasets collected over many years, opening the door to more in-depth analysis.

This study presents not only a technical means for AMS identification using asynchronous data but also designs and executes a scalable data collection infrastructure that can meet the requirements of an extensive bridge health monitoring platform. In the framework depicted in Fig. 6, our designed smartphone application contains a registry of bridges with user-defined geo-fences. Smartphones automatically collect vibration data when entering the geo-fences, package it, and save it on cloud storage. A backend server is triggered at a user-defined time cadence to consume stored data, split and preprocess them for each bridge, and run the proposed algorithm to identify current estimations of AMSs. This automatic and recurring process provides a time series of identified real-time dynamic properties of each bridge separately, which is key to real-time bridge monitoring and a foundation for a data-driven alarm system. The time series are closely tracked for any anomalies attributed to structural damage or condition changes. Once an abnormal change is observed, bridge owners can be alerted automatically to orchestrate an onsite inspection.

This framework is readily applicable to bridge networks in urban and suburban areas with high smartphone penetration rates, in which there are large, pre-existing mobile sensor datasets that can be re-purposed for bridge monitoring. The AADT over Delaware Water Gap Bridge in DE, USA, was nearly 50,000 in 2022. If only a mere 1% of vehicles participated in smartphone data collection, about 500 datasets would be generated daily. The same cars that collect data on this bridge may continue on I-80 interstate highway and contribute data to other bridges. This example illustrated how existing human mobility mechanisms can drive regular data generation for a large population of bridges. Commercial and municipal vehicle fleet services, e.g., ridesourcing companies, or public works, have the unique potential to support and distributed data collection.

This study elaborates on one specific application: tracking AMSs over time to identify structural condition abnormalities as a feasible solution for cost-effective widespread monitoring. However, the massive amount of data in the long run of the data collection framework is by no means limited to this specific downstream use case. By accumulating more data with more diversified environmental/road type/bridge structure catalogs, we provide a rich source of data for more specific downstream tasks such as damage localization and/or more holistic final outputs such as remaining-life analysis of structures.

### Continuous monitoring simulation

**Fig. 7:**
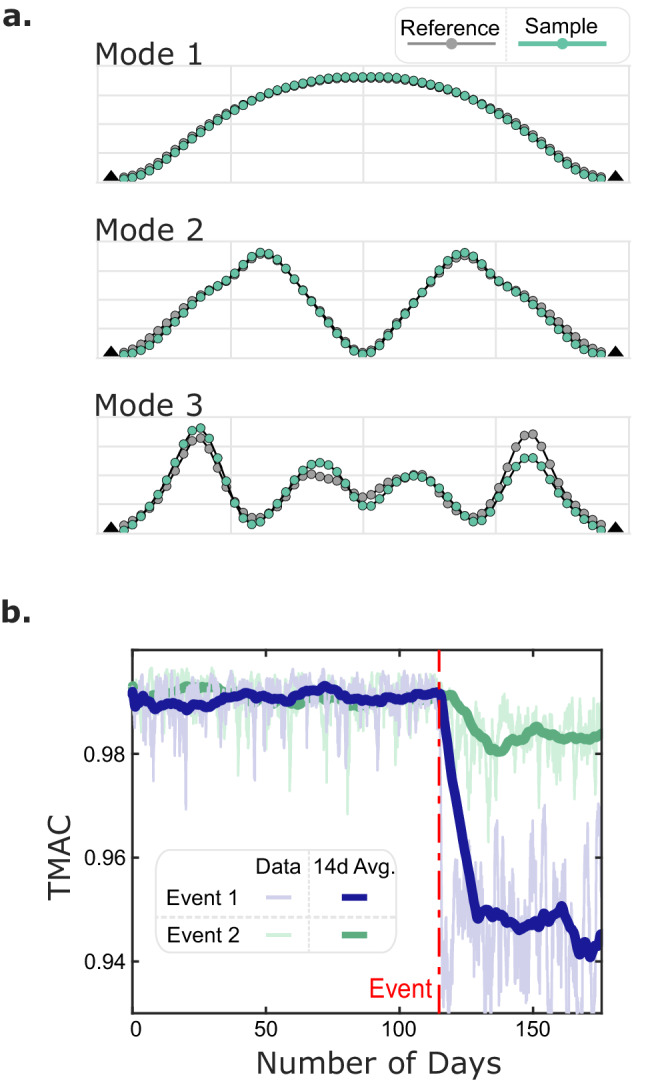
Simulation of a mobile sensing monitoring campaign on a bridge that experiences damage. **a** The first three AMS estimates, with the ‘reference’ being an estimate from mobile sensors prior to damage compared to a ‘sample’ after the damage. **b** Graph of TMAC overtime where the reference AMS is taken before day zero compared to samples taken on a daily basis.

Once the bridge dynamic properties have been estimated with the proposed method, the monitoring data become compatible with a data-driven condition assessment. For descriptive purposes, we simulated a monitoring campaign of a 59m truss bridge that undergoes two common damage cases: element stiffness change, which implies the occurrence of common damage types such as corrosion or fatigue in the element, and differential support settlement^39,67^. In addition, the effects of environmental factors, such as temperature, were ignored for simplicity, and integrating mobile sensing into more comprehensive damage identification frameworks is of interest for future works. The goal was to detect the changes in structural condition by only using signals collected by passing vehicles based on AMS estimates (Details of the simulation and damage detection method are in the “Methods” section). Damage detection algorithms generally follow the same process of collecting data, estimating dynamic properties, and converting estimates to a damage-sensitive index. Here, the index for tracking the structural health is the total modal assurance criteria (TMAC)^68^, essentially a similarity metric comparing AMS values estimated at different points in time. The first three modes (comparable to the GGB case study) were included in the TMAC calculation for this analysis, but this is generally a parameter that depends on the number of identified modes. Figure 7 shows when a batch incorporates the SVTs generated before and after the event (defect), the TMAC values drop with statistical significance. This drop means the changes in the mode shape are detectable and non-transitory, which differ from variations in the underlying state of the structure. The output of readily available damage detection methods, such as change point analysis^69^, can be configured to function as an early alarm system that immediately notifies bridge stakeholders. This simulation demonstrates how the estimated AMS can be incorporated into existing damage detection methods. The damage cases considered here are only illustrative and are far from exhaustive. As with all damage detection methods, the sensitivities of the damage-sensitive features and setup of statistical models will vary with damage case and damage severity, and can ultimately limit detectability. Additional work needs to be done to create a robust platform for monitoring that incorporates not only AMS but a wide range of information.

## Discussion

Four studies in this paper demonstrated a wide range of real-world examples in which important spatial vibration characteristics of bridges, i.e., absolute value mode shapes (AMSs), can be extracted successfully from crowdsourced smartphone-vehicle trip (SVT) data. Bridges from three general classes were considered: short-span, medium-to-long-span, and long-span, and three types of crowdsourced SVT datasets were used: controlled, partially controlled, and uncontrolled.

The successful applications on the Golden Gate Bridge (GGB) show capabilities for identifying AMSs of long-span bridges in both vertical and horizontal directions (see Fig. 3a–e). Bridges with long main spans (>500 m) belong to a small yet special category. While there are fewer than 100 such bridges in the world, they have high structural flexibility tied to low modal frequencies (i.e., several modes below 1 Hz^63,70^). This is notable for the case of SVT data as this frequency range is distinctly below the typical frequency range of vehicle suspension systems (1−3 Hz). Thus, the adverse effects of vehicle-bridge-road interaction are mitigated. In addition, SVT datasets collected on long-span bridges will have a larger sample size for a given speed and sampling rate. Finally, long-span bridges are among the most traveled, as measured by large AADT values, facilitating high-rate SVT data streams. In summary, with the potential for large volumes of high-quality datasets, SVT data may present their highest value to long-span bridges.

The studies on the Cadore Bridge (CAD) and Gene Hartzell Memorial Bridge (GHMB) confirmed the method’s applicability to a larger group of existing bridges with maximum spans between 50 and 500 m, i.e., medium- to long-span bridges. According to the national bridge inventory (NBI) curated by the Federal Highways Administration (FHWA), about 12,000 bridges (2%) in the US belong to this class. Similarly, the successful study on the short-span Ciampino Bridge (CMP) represents the applicability of the method to US bridges having maximum spans between 15 and 50 m, which includes nearly 163,000 bridges (26%). Even though our cases were diverse, by span length, they only represent about a quarter of all bridges, and additional work needs to be done to discover the limitations of the applicability of the method.

From a structural dynamics perspective, bridges with shorter spans are stiffer than long-span bridges and are more likely to possess some modal frequencies between the typical vehicle band 1−3 Hz. Note that the modal frequencies for both the second horizontal mode for CAD (see CAD-PC-H2 in Fig. 3g) and the third vertical mode for GHMB (see GHMB-C-V3 in Fig. 3f) fall within this range and are therefore subject to high noise from vehicle-bridge-road interaction. This methodology does not include modeling of the vehicle system nor any processes for decoupling vehicle dynamics, e.g., empirical mode decomposition, deconvolution, etc.^71^; nonetheless, accurate AMSs were extracted, which may suggest that large and diverse SVT datasets, acquired using many different vehicles, help mitigate negative interaction effects. Notably, only one AMS was successfully extracted from each of these bridges. This result is partially dictated by the random nature of the traffic excitation: there is no guarantee that a given mode is sufficiently active in the measured structural response at the space-time coordinates of the SVT data. Large data volumes (on the order of thousands or hundreds of thousands) would dramatically improve the odds that individual structural modes have a strong presence in aggregate space-frequency maps. Generally, for robust damage detection, several bridge modes should be consistently tracked over time.

The AMSs are directly linked to reliable indicators of structural damage, such as mode shape curvature, total modal assurance criteria (TMAC), etc.^68^, and thereby establish a fundamental functionality for a damage detection system. The robust simulation analyses in exemplify two crowdsensing scenarios in which these features are used to detect the presence of bridge damage. A 60-m-long bridge was considered with a mobile sensor network that produced 100 SVT datasets per day over the course of six months. The identified AMSs for three modes were used to track the TMAC value over time (days) which led to accurate damage detection within a few days of the damage event. While this analysis was successful in establishing fundamental damage detection capabilities based on crowdsourced mobile smartphone data, further research is needed to quantify sensitivities of AMS-based features to structural damage and evaluate robustness using data from real-world bridge damage cases.

The approach proposed in this paper is based on statistical signal processing techniques and does not utilize machine learning (ML). Over the past decade, applications of data-driven ML methods have initiated enormous advances in the sciences, broadly shaping fields such as physics, computer vision, robotics, natural language processing, neuroscience, etc.^72–75^, with growing applications in civil engineering^48,76–78^. That is, the results here establish the initial, known capabilities for extracting spatial vibration characteristics features but may underreport the extent of dynamic features and accuracies that are possible for the considered datasets. For instance, in the studies presented, the accuracy of the results from SVT data was evaluated through a direct comparison with results based on traditional fixed sensor networks, while trained AI models may enable techniques for automated cross-validation of modal properties.

Bridge condition monitoring based on SVT data leverages existing vehicle mobility networks and high smartphone penetration rates. These approaches offer very low upfront monitoring costs compared to traditional sensing systems and enable a scalable information retrieval process: all bridges that are covered by the smartphone-vehicle network can be analyzed using these tools. Urban bridges generally have high AADT levels, such that low participation rates can still yield ample data streaming volumes, e.g., 1% of the AADT of the Golden Gate Bridge amounts to over 1000 daily trips. Conversely, data streaming volumes for bridges in rural areas may be limited by comparatively lower AADT values; yet, widespread and long-term datasets combined with advances in transfer learning could help utilize prior analyses of similar bridges to enable dynamic property estimation using sparse SVT data.

A key ambition for crowdsourcing infrastructure condition information is the creation of an open database of bridge vibration records, curated with historic dynamic property estimates, bridge model files, and design details such as material or span, etc. The overarching goal is to provide bridge owners and inspectors an opportunity to focus on key, pre-identified bridge locations or components of interest, to enable more frequent, more quantitative inspections, and to support preventative maintenance protocols. Such a database would complement existing archives, e.g., the National Bridge Inventory, which is managed by the FHWA, with the addition of maintaining historical data and records. Regular observations of structural behavior over a long term (over 1 year) are essential to establishing baselines and performance benchmarks needed for condition evaluations^79^. Centralization and standardization of archival vibration data and structural health reports would be critical to the success and impact of this program. First, widespread coverage of bridges would promote the participation of bridge owners. Second, standardized and curated datasets assist with the rapid development and application of new techniques, such as developing trained AI models for condition monitoring^80^ or alternative damage index metrics^81^.

The establishment of a mobile sensing platform of this scale creates new interdisciplinary challenges. Everyday use and reliability have substantial computing, network, and cloud infrastructure needs for which existing cellular networks and smartphone ownership levels provide an important foundation. There are a very large number of eligible SVT datasets that extensively cover US infrastructure and have already been collected by ridesharing companies and apps such as Uber, Lyft, Waze, etc. Figure 3b shows a successful implementation based on a small number of uncontrolled SVT trips collected by Uber. There is high potential value within these enormous, pre-existing datasets, which present opportunities for ridesharing businesses to collaborate with municipalities and state Departments of Transportation. Advances in smart vehicle technologies, e.g., vehicle-to-infrastructure communication, could make a large positive impact by accelerating data transmission, storage, and analysis. Developments in material science can help connect sparse structural response measurements to local and global behavior^82^, e.g., load distributions^83^, multi-scale analysis and design^84^. The concept of an open database of bridge records poses potential legal constraints and questions which will depend on jurisdiction. Is there a need to restrict database access to a limited number of users, organizations, or institutions for national security? Would there be a there a legal obligation for a bridge owner to act on data-driven reports describing significant indicators of severe structural damage?

The findings of this paper could have a substantial impact on the monitoring and management of bridges globally, through transforming costly sensory (thus seemingly luxury) information on a small subset of the bridge population into a distributed, accessible, and affordable commodity. The tools developed are broadly applicable to all bridges; yet, there remains a need to continue to study the accuracy and efficacy of using SVT data in additional scenarios to better identify the strengths and potential limitations of the approach for existing bridges. The results presented in Fig. 7 emphasize the power of regularly collecting and analyzing SVT data over time for damage detection and support the integration of low-cost monitoring as a standard into bridge management practices.

## Methods

### Automated smartphone data collection and processing

An Android smartphone application named Good Vibrations was developed to record vibrations, locations, and orientations data generated when a smartphone is moving over a monitored infrastructure. The main objectives of this smartphone application can be summarized as follows:

1. Detect when a smartphone is entering or leaving a monitored area using geofences.
2. Record and store the data coming from the sensors of the smartphone at the highest available frequency.
3. Minimize the energy consumption of the application.

However, the first and last items of this list are generally in contrast. On one hand, to detect the position of a smartphone with a good level of accuracy while tracking its movements, it is necessary to keep the GPS receiver always ON; on the other hand, an active GPS receiver can consume most of the battery of a smartphone. To solve this issue, we decided to implement a multi-barrier activation approach.

The first activation barrier consisted of detecting if the user was currently driving. When the application is active, a service listens for an activity change event generated by the Android OS. Activity detection is a service provided by the Android OS which uses an integrated machine learning model to detect whether the smartphone is standing still or carried by a user who is walking, biking, or driving by just analyzing the signal generated by the IMU sensors of the smartphones. This approach is more energy efficient than other localization methods at the cost of a small activation delay. The second activation barrier occurs when the user is driving close to a monitored infrastructure. In this case, the application relies on the GPS receiver of the smartphone, and it implements the following logic to detect if the user is close: If a monitored infrastructure is reachable in less than 60 s, the GPS receiver is left ON, and the current location of the device is collected every 5 s. Otherwise, the application will turn off the GPS module and will schedule a new activation of the GPS receiver in *t* s. This *t* value is computed based on the distance from the closest infrastructure.

The *t* value is a very conservative estimate, yet preliminary experiments indicated a good trade-off between accuracy, energy consumption, and computation capabilities. Finally, if the user is driving, the location and speed reported by the GPS receiver indicate that a monitored infrastructure is reachable in less than 30 s, and the accuracy provided by the GPS receiver is less than 10 m the recording process can start. The data generated by the GPS receiver, (timestamp, latitude, longitude, speed, accuracy) the rotation vector (timestamp and rotation quaternion) and the accelerometer (timestamp and x, y, z components of the acceleration vector) are recorded independently at the maximum sample rate allowed by the device. The accuracy of the GPS data was measured by “GPS error” in the cases of the Uber and GoodVibrations apps (uncontrolled ridesourcing and partially controlled datasets). The SensorPlay app did not provide any metrics for GPS accuracy; Golden Gate Bridge GPS errors averaging around 7.7 m and Ciampino the average GPS error was about 4.3 m. Generally, GPS data is recorded every second, while Accelerations and Rotation data rate can vary depending on the specific device between 50 and 500 Hz.

To allow for the fastest possible recording, these data are initially stored in memory and then moved to the storage of the device when the data collection is over (or periodically to avoid filling up the memory). To avoid losing measurements during the memory off load, the entire collection process is multithreaded and based on synchronization queues in a producer-consumer fashion. The new data is inserted into dedicated queues (one for each sensor) and a writer thread is responsible for moving the data from the queues to the physical storage of the device when required. This minimizes the amount of code executed as a response to a new data sample, while leaving the data in terms of timestamps and values exactly as provided by the OS. Once the user leaves the monitored area, a first check on the GPS trace is performed. However, a driver could get really close to an infrastructure without crossing it. To filter out these cases the application checks that the recorded GPS trajectory intersects a set of checkpoints located on the bridge. If this happens, the scan is ready for uploading, otherwise the scan is discarded. Before uploading the collected scans to the Cloud, the data is compressed and divided into smaller chunks to ease the upload process. Finally, when the smartphone is connected to the Internet the scans are uploaded to the Cloud and if the process is successful the uploaded data is removed from the smartphone.

### Absolute mode shape identification methodology

The algorithm detects the change in magnitude of known modal frequencies with the position on the bridge, which is proportional to the AMS. For data collection, vehicles carry smartphones that take acceleration and location measurements. Since the signals are collected within moving vehicles, the bridge vibrations are highly contaminated by the road profile and vehicle dynamics. Furthermore, the low-quality smartphone sensors introduce additional sampling errors and measurement noise. For these reasons, the bridge vibrations are almost imperceptible in a single signal, however, by averaging many trips the spatial characteristics of the vibrations appear. The averaging scheme is presented in Table 1 and summarized below.

**Table 1:** Summary of the core methodology used for identifying absolute natural mode shapes

1. Depending on the axis of interest, corresponding raw acceleration signals ${a}_{i}^{r}(t)$ are collected with smartphones: *i* = 1, 2, …, *N*.
2. Preprocessing: ${a}_{i}^{r}(t)$ is downsampled to a desired frequency range, yielding *a*_*i*_(*t*). The downsampled signals are checked for stationarity using augmented Dickey-Fuller test^88^ to filter out corrupted signals with baseline shifts or unreasonable variations.
3. (Optional) Depending on the direction of the vehicle motion (right-left or left-right) and in case that the inspected span is surrounded by expansion joints, a fractual value *α* ∈ [0, 0.2] is selected and the initial portions of the signals are trimmed: *a*_*i*_(*t*) ≔ *a*_*i*_(*t*_*n*_): *n* > *α* × ∣*a*_*i*_(*t*)∣.
4. Synchrosqueezed wavelet transform is calculated using *a*_*i*_(*t*), resulting ${T}_{{a}_{i}}(f,t)$, in which 0 ≤ *f* ≤ *f*_*Nyq*_ and *f*_*Nyq*_ is the Nyquist frequency defined by the sampling rate.
5. ${T}_{{a}_{i}}(f,t)$ is mapped into spatial coordinates using available time-stamped GPS coordinates (provided by the app), resulting ${T}_{{a}_{i}}(f,x)$ in which *x* ∈ [0, *L*_*br*_] is the longitudinal position on the bridge with total length of *L*_*br*_.
6. Select values for bandwidth *ϵ*, spatial segment width Δ, and a spatial stride *S*.
7. Given a desired natural frequency *f*_*k*_, a series of frequency-space grids are defined as follows:
$${B}_{ij}={T}_{{a}_{i}}([{f}_{k}-\epsilon /2,{f}_{k}+\epsilon /2],[j\times S,\max \{{L}_{br},\Delta +j\times S\}])$$ for *j* = 1, …, ⌊(*L*_*br*_ − Δ)/*S*⌋ + 1.
*B*_*ij*_ contains wavelet amplitudes for signal *a*_*i*_(*t*) in a *j*th frequency-space grid. If ∣*B*_*ij*_∣ = 0, it is skipped.
8. The mean of amplitudes over all signals for each frequency-space grid is calculated: ${\mu }_{j}=\frac{1}{N}\mathop{\sum }_{1}^{N}p:p\in {B}_{ij}$.
9. Finally, an ordered set of *μ*_*j*_ for *j* = 1, …, ⌊(*L*_*br*_ − Δ)/*S*⌋ + 1 presents the aggregated absolute spatial pattern of the bridge on frequency *f*_*k*_. For a fair choice of *f*_*k*_, this spatial signature should converge to the absolute natural mode shape.

By inspecting downsampled signals, we notice that some samples contain exorbitant baseline drift or offsets. Such erroneous samples can be treated in two ways: high-pass filtering or discarding. Due to the low resolution of the smartphone accelerometers, the latter approach seems more reasonable. In other words, a large baseline drift masks all higher frequency contents in a low-resolution signal and therefore, filtering is ineffective. In this study, an optional preprocessing step is introduced to automatically detect and skip extremely nonstationary signals. The augmented Dickey-Fuller test is performed on the signal and the test statistic value is checked with a user-defined threshold and surpassing values point to a rejection decision.

Consistent spatial features that cause impulses in the collected acceleration time series will affect the entire frequency spectrum and skew the spatial average in the AMS identification process. Since the road profile is unknown, this is a difficult obstacle to overcome and additional work in signal processing or trip filtering needs to be done. However, through tests on multiple bridges, we noticed there are consistent impulses in the acceleration time series upon entry and exit of the bridges due to the expansion joints. For this reason, we recommend trimming the beginning of signals to allow the vehicle vibrations from the impulse to dampen.

The aggregation process spatially averages frequency contents to determine the relative magnitude differences at each location on the bridge. To achieve this, the bridge is divided into overlapping segments; the width, Δ, and segment centers distance, S, are two parameters of the method. The synchrosqueezed wavelet transform converts each acceleration time series to the time-frequency domain, and the instantaneous magnitudes are calculated. Lastly, averaging the magnitudes in each segment, *B*_*ij*_, at the modal frequencies of the bridge for all trips yields the AMS. Including a small bandwidth, *ϵ*, around the modal frequency in the averaging process leads to robust results on noisy datasets.

The method relies on the prior knowledge of the modal frequencies, and for this study, the natural frequencies are determined in two different ways. First, the frequencies are found using a traditional fixed sensor network, the state of practice in SHM, which were used as a reference for analysis. Second, the natural frequencies are found with the recently proposed algorithm MPMF^36^. The goal of determining the frequencies in such a way is to demonstrate that all of the results in the work can be found only using measurements obtained from passing vehicles. System identification analyses for all bridges are discussed in Supplementary Note 3.

### Bridge condition monitoring simulation

The goal of the simulation is to mimic the long-term monitoring of a bridge with mobile sensors, and detect system changes. The case study is a steel truss bridge in Japan with a main span of 59.2 m. A 3D finite element (FE) model is generated in SAP2000 and the accuracy of the model is validated by matching the operational modal characteristics with those found from real-world instrumentation^85^, see Table S1.

In the FE model, two common and general damage scenarios are considered: (a) a cross-sectional reduction in a critical element, and (b) differential settlement on a terminal support. During testing, the simulation was repeated for varying degrees of severity of settlements and element stiffness reductions. Figure S1a denotes the truss element and support where the damages were imparted. In the analysis presented, the element stiffness change was created by a cross-sectional area reduction of 50%, and for the differential support settlement, the support was moved down by 10 cm.

Artificial SVTs were generated following a simplified vehicle bridge interaction procedure^86^. In summary, this simulation method assumes the masses of the vehicles are negligible compared to the mass of the bridge, and instead of modeling the physical interactions, the excitation is assumed to be random and gaussian. The excitation was modeled with random impulses applied at nodes along the span. SVT trips are created by taking the bridge response of the bridge at the location of the vehicle and adding it to the road profile at that location. This signal is used as an input to a quarter-car model. The accelerations of the sprung mass are used as the artificial SVTs in the analysis. Vehicles traversed the bridge at varying speeds from 2 to 9 m/s and with Gaussian measurement noise with SNR of five. The low value for SNR aims to replicate the noisy measurement of the mobile sensors.

The rate of SVT collected over a fixed time horizon is an important factor as it influences the time needed by the algorithm to detect potential changes in the estimated parameters and generally; i.e., a large number of trips in a single day results in a fast identification of the changes compared to a bridge with only a few passages per day. For this analysis, we have considered that it is possible to record 100 trips per day for the simulated bridge. The annual average daily traffic of bridges in the United States, depending on the rural or urban location of the bridge, is usually more than 10,000 vehicles per day. For instance, for Golden Gate bridge and Gene Hartzell Memorial bridge, which are among the case studies of the present research, the annual daily average is approximately 88,000 and 59,000 vehicles per day, respectively. So, even if a small fraction of these vehicles are capable of collecting data, it would not be significantly different from our assumption.

### Supplementary information

Supplementary Material

## Data Availability

All data used to create Fig. 3 is available for download on Dryad (10.5061/dryad.15dv41p49) as “Commodifying infrastructure spatial dynamics with crowdsourced smartphone data,” and if used, must be cited as seen here^87^.

## Code availability

Code for this experiment will be available on Dryad (10.5061/dryad.15dv41p49) as a Jupyter Notebook, and is formatted to be used in Google Colab for ease of implementation. The code reproduces the Golden Gate Bridge Results for the controlled case.

## References

[1] American Society of Civil Engineers. *Failure to Act: Closing the Infrastructure Investment Gap for America’s Economic Futur*e (American Society of Civil Engineers, 2016).

[2] Koks EE (2019). A global multi-hazard risk analysis of road and railway infrastructure assets. Nat. Commun..

[3] Van Aalst MK (2006). The impacts of climate change on the risk of natural disasters. Disasters.

[4] Mitchell JF, Lowe J, Wood RA, Vellinga M (2006). Extreme events due to human-induced climate change. Philos. Trans. R. Soc. A: Math., Phys. Eng. Sci..

[5] Banholzer, S., Kossin, J. & Donner, S. in *Reducing Disaster: Early Warning Systems for Climate Change*, 21–49 (Springer, 2014).

[6] Cook W, Barr PJ, Halling MW (2015). Bridge failure rate. J. Perform. Constr. Facil..

[7] Van Leeuwen, Z. & Lamb, R. *Flood and Scour Related Failure Incidents at Railway Assets Between 1846 and 2013* (Railway Safety & Standards Board, 2014).

[8] Garg RK, Chandra S, Kumar A (2022). Analysis of bridge failures in India from 1977 to 2017. Struct. Infrastruct. Eng..

[9] Jeong Y, Kim W, Lee I, Lee J (2018). Bridge inspection practices and bridge management programs in China, Japan, Korea, and US. J. Struct. Integr. Maint..

[10] Lynch JP (2007). An overview of wireless structural health monitoring for civil structures. Philos. Trans. R. Soc. A: Math., Phys. Eng. Sci..

[11] Farrar, C. R. & Worden, K. *Structural Health Monitoring: A Machine Learning Perspective* (John Wiley & Sons, 2012).

[12] Sanayei M, Khaloo A, Gul M, Catbas FN (2015). Automated finite element model updating of a scale bridge model using measured static and modal test data. Eng. Struct..

[13] Khaloo A, Lattanzi D, Cunningham K, Dell’Andrea R, Riley M (2018). Unmanned aerial vehicle inspection of the placer river trail bridge through image-based 3d modelling. Struct. Infrastruct. Eng..

[14] Momtaz Dargahi M, Khaloo A, Lattanzi D (2022). Color-space analytics for damage detection in 3d point clouds. Struct. Infrastruct. Eng..

[15] Lin C, Yang Y (2005). Use of a passing vehicle to scan the fundamental bridge frequencies: an experimental verification. Eng. Struct..

[16] Feng M, Fukuda Y, Mizuta M, Ozer E (2015). Citizen sensors for SHM: use of accelerometer data from smartphones. Sensors.

[17] Yang Y-B, Lin C, Yau J (2004). Extracting bridge frequencies from the dynamic response of a passing vehicle. J. Sound Vib..

[18] Yang Y, Chang K (2009). Extracting the bridge frequencies indirectly from a passing vehicle: parametric study. Eng. Struct..

[19] Siringoringo DM, Fujino Y (2012). Estimating bridge fundamental frequency from vibration response of instrumented passing vehicle: analytical and experimental study. Adv. Struct. Eng..

[20] Zhang Y, Wang L, Xiang Z (2012). Damage detection by mode shape squares extracted from a passing vehicle. J. Sound Vib..

[21] Yang, Y.-B., Yang, J. P., Zhang, B. & Wu, Y. *Vehicle Scanning Method for Bridges* (Wiley Online Library, 2020).

[22] Sitton JD, Rajan D, Story BA (2020). Bridge frequency estimation strategies using smartphones. J. Civ. Struct. Health Monit..

[23] Sitton JD, Zeinali Y, Rajan D, Story BA (2020). Frequency estimation on two-span continuous bridges using dynamic responses of passing vehicles. J. Eng. Mech..

[24] Marulanda J, Caicedo JM, Thomson P (2016). Modal identification using mobile sensors under ambient excitation. J. Comput. Civ. Eng..

[25] Matarazzo TJ, Pakzad SN (2016). Structural identification for mobile sensing with missing observations. J. Eng. Mech..

[26] Matarazzo TJ, Pakzad SN (2016). Truncated physical model for dynamic sensor networks with applications in high-resolution mobile sensing and bigdata. J. Eng. Mech..

[27] Matarazzo TJ, Pakzad SN (2018). Scalable structural modal identification using dynamic sensor network data with STRIDEX. Comput.-Aided Civ. Infrastruct. Eng..

[28] Malekjafarian A, OBrien EJ (2014). Identification of bridge mode shapes using short time frequency domain decomposition of the responses measured in a passing vehicle. Eng. Struct..

[29] Ozer E, Feng MQ, Feng D (2015). Citizen sensors for SHM: towards a crowdsourcing platform. Sensors.

[30] Ozer E, Purasinghe R, Feng MQ (2020). Multi-output modal identification of landmark suspension bridges with distributed smartphone data: Golden gate bridge. Struct. Control Health Monit..

[31] Figueiredo E, Moldovan I, Alves P, Rebelo H, Souza L (2022). Smartphone application for structural health monitoring of bridges. Sensors.

[32] McGetrick P, Hester D, Taylor S (2017). Implementation of a drive-by monitoring system for transport infrastructure utilising smartphone technology and GNSS. J. Civ. Struct. Health Monit..

[33] Matarazzo TJ (2018). Crowdsensing framework for monitoring bridge vibrations using moving smartphones. Proc. IEEE.

[34] Eshkevari SS, Cronin L, Matarazzo TJ, Pakzad SN (2023). Bridge modal property identification based on asynchronous mobile sensing data. Struct. Health Monit..

[35] Quqa S, Giordano PF, Limongelli MP (2022). Shared micromobility-driven modal identification of urban bridges. Autom. Constr..

[36] Matarazzo TJ (2022). Crowdsourcing bridge dynamic monitoring with smartphone vehicle trips. Commun. Eng..

[37] OKeeffe KP, Anjomshoaa A, Strogatz SH, Santi P, Ratti C (2019). Quantifying the sensing power of vehicle fleets. Proc. Natl Acad. Sci. USA.

[38] Askegaard, V. & Mossing, P. *Long Term Observation of Rc-bridge Using Changes in Natural Frequency. Nordic Concrete Research. Publication No 7* (Nordic Concrete Federation, 1988).

[39] Peeters B, De Roeck G (2001). One-year monitoring of the Z24-bridge: environmental effects versus damage events. Earthq. Eng. Struct. Dyn..

[40] Peeters B, Maeck J, De Roeck G (2001). Vibration-based damage detection in civil engineering: excitation sources and temperature effects. Smart Mater. Struct..

[41] Liang Y, Li D, Song G, Feng Q (2018). Frequency co-integration-based damage detection for bridges under the influence of environmental temperature variation. Measurement.

[42] Ralbovsky M, Deix S, Flesch R (2010). Frequency changes in frequency-based damage identification. Struct. Infrastruct. Eng..

[43] Kim J-T, Park J-H, Lee B-J (2007). Vibration-based damage monitoring in model plate-girder bridges under uncertain temperature conditions. Eng. Struct..

[44] Jin C, Jang S, Sun X, Li J, Christenson R (2016). Damage detection of a highway bridge under severe temperature changes using extended Kalman filter trained neural network. J. Civ. Struct. Health Monit..

[45] Fan W, Qiao P (2011). Vibration-based damage identification methods: a review and comparative study. Struct. Health Monit..

[46] Farrar C, James Iii G (1997). System identification from ambient vibration measurements on a bridge. J. Sound Vib..

[47] Shi Z, Law S, Zhang L (2000). Damage localization by directly using incomplete mode shapes. J. Eng. Mech..

[48] Lee JJ, Lee JW, Yi JH, Yun CB, Jung HY (2005). Neural networks-based damage detection for bridges considering errors in baseline finite element models. J. Sound Vib..

[49] Hu C, Afzal MT (2006). A statistical algorithm for comparing mode shapes of vibration testing before and after damage in timbers. J. Wood Sci..

[50] OBrien EJ, Malekjafarian A (2016). A mode shape-based damage detection approach using laser measurement from a vehicle crossing a simply supported bridge. Struct. Control Health Monit..

[51] Liew KM, Wang Q (1998). Application of wavelet theory for crack identification in structures. J. Eng. Mech..

[52] Hong J-C, Kim Y, Lee H, Lee Y (2002). Damage detection using the Lipschitz exponent estimated by the wavelet transform: applications to vibration modes of a beam. Int. J. Solids Struct..

[53] Douka E, Loutridis S, Trochidis A (2004). Crack identification in plates using wavelet analysis. J. Sound Vib..

[54] Chang C-C, Chen L-W (2005). Detection of the location and size of cracks in the multiple cracked beam by spatial wavelet based approach. Mech. Syst. Signal Process..

[55] Poudel UP, Fu G, Ye J (2007). Wavelet transformation of mode shape difference function for structural damage location identification. Earthq. Eng. Struct. Dyn..

[56] Tan, C., Elhattab, A. & Uddin, N. Wavelet based damage assessment and localization for bridge structures. In *26th ASNT Research Symposium*, 228–240 (2017).

[57] Pandey A, Biswas M, Samman M (1991). Damage detection from changes in curvature mode shapes. J. Sound Vib..

[58] Wahab MA, De Roeck G (1999). Damage detection in bridges using modal curvatures: application to a real damage scenario. J. Sound Vib..

[59] Kim BH, Park T, Voyiadjis GZ (2006). Damage estimation on beam-like structures using the multi-resolution analysis. Int. J. Solids Struct..

[60] Feng D, Feng MQ (2016). Output-only damage detection using vehicle-induced displacement response and mode shape curvature index. Struct. Control Health Monit..

[61] Shokrani Y, Dertimanis VK, Chatzi EN, N. Savoia M (2018). On the use of mode shape curvatures for damage localization under varying environmental conditions. Struct. Control Health Monit..

[62] Allemang, R. J. A correlation coefficient for modal vector analysis. In *Proc. 1st Int. Modal Analysis Conference*, 110–116 (1982).

[63] Pakzad SN, Fenves GL (2009). Statistical analysis of vibration modes of a suspension bridge using spatially dense wireless sensor network. J. Struct. Eng..

[64] Abdel-Ghaffar AM, Scanlan RH (1985). Ambient vibration studies of Golden Gate Bridge: I. Suspended structure. J. Eng. Mech..

[65] Çelebi M (2012). Golden Gate Bridge response: a study with low-amplitude data from three earthquakes. Earthq. Spectra.

[66] Malekjafarian A, OBrien EJ (2017). On the use of a passing vehicle for the estimation of bridge mode shapes. J. Sound Vib..

[67] Riasat Azim M, Gül M (2020). Damage detection of steel-truss railway bridges using operational vibration data. J. Struct. Eng..

[68] Gao Y, Spencer B (2002). Damage localization under ambient vibration using changes in flexibility. Earthq. Eng. Eng. Vib..

[69] Killick R, Fearnhead P, Eckley IA (2012). Optimal detection of changepoints with a linear computational cost. J. Am. Stat. Assoc..

[70] Chen, W.-F. & Duan, L. *Handbook of International Bridge Engineering* (CRC Press, 2013).

[71] Eshkevari SS, Matarazzo TJ, Pakzad SN (2020). Bridge modal identification using acceleration measurements within moving vehicles. Mech. Syst. Signal Process..

[72] Jordan MI, Mitchell TM (2015). Machine learning: trends, perspectives, and prospects. Science.

[73] Marx V (2013). The big challenges of big data. Nature.

[74] He, K., Zhang, X., Ren, S. & Sun, J. Deep residual learning for image recognition. In *Proceedings of the IEEE Conference on Computer Vision and Pattern Recognition*, 770–778 (2016).

[75] Brunton SL, Proctor JL, Kutz JN (2016). Discovering governing equations from data by sparse identification of nonlinear dynamical systems. Proc. Natl Acad. Sci. USA.

[76] Spencer Jr BF, Hoskere V, Narazaki Y (2019). Advances in computer vision-based civil infrastructure inspection and monitoring. Engineering.

[77] Khaloo A, Lattanzi D, Jachimowicz A, Devaney C (2018). Utilizing UAV and 3D computer vision for visual inspection of a large gravity dam. Front. Built Environ..

[78] Rafiei MH, Adeli H (2018). A novel unsupervised deep learning model for global and local health condition assessment of structures. Eng. Struct..

[79] Smith IF (2016). Studies of sensor data interpretation for asset management of the built environment. Front. Built Environ..

[80] Malekjafarian A, Golpayegani F, Moloney C, Clarke S (2019). A machine learning approach to bridge-damage detection using responses measured on a passing vehicle. Sensors.

[81] Liu, J. et al. Damage-sensitive and domain-invariant feature extraction for vehicle-vibration-based bridge health monitoring. In *ICASSP 2020-2020 IEEE International Conference on Acoustics, Speech and Signal Processing (ICASSP)*, 3007–3011 (IEEE, 2020).

[82] Feng, B. T., Ogren, A. C., Daraio, C. & Bouman, K. L. Visual vibration tomography: Estimating interior material properties from monocular video. In *Proceedings of the IEEE/CVF Conference on Computer Vision and Pattern Recognition*, 16231–16240 (2022).

[83] Guo K, Buehler MJ (2020). A semi-supervised approach to architected materials design using graph neural networks. Extrem. Mech. Lett..

[84] Nadkarni N, Daraio C, Kochmann DM (2014). Dynamics of periodic mechanical structures containing bistable elastic elements: from elastic to solitary wave propagation. Phys. Rev. E.

[85] Chang K-C, Kim C-W (2016). Modal-parameter identification and vibration-based damage detection of a damaged steel truss bridge. Eng. Struct..

[86] Sadeghi Eshkevari S, Matarazzo TJ, Pakzad SN (2020). Simplified vehicle–bridge interaction for medium to long-span bridges subject to random traffic load. J. Civ. Struct. Health Monit..

[87] Cronin, L. et al. *Commodifying Infrastructure Spatial Dynamics with Crowdsourced Smartphone Data* (2024).

[88] Dickey DA, Fuller WA (1979). Distribution of the estimators for autoregressive time series with a unit root. J. Am. Stat. Assoc..

