# Supplementary material for: Bridging the Gap: commodifying infrastructure spatial dynamics with crowdsourced smartphone data
Source: Commun Eng. 2024 Jul 4;3:93. doi: 10.1038/s44172-024-00243-y (PMC11224296; doi:10.1038/s44172-024-00243-y)
Supplement: Supplementary file 1 — Supplementary Material [file 44172_2024_243_MOESM1_ESM.pdf]

Supplementary Materials for

Bridging the Gap: Commodifying Infrastructure Spatial Dynamics with Crowdsourced Smartphone Data

Liam Cronin, Soheil Sadeghi Eshkevari, Thomas J. Matarazzo, Sebastiano Milardo, Iman Dabbaghchian, Paolo Santi, Shamim N. Pakzad, Carlo Ratti

Correspondence to: lmc219@lehigh.edu

This PDF file includes:

Supplementary Text
Figure S 1 to S 5
Tables S 1

Supplementary Note 1: Additional Information for Smartphone Vehicle Trip (SVT) Data Collection

Gene Hartzell Memorial Bridge

The data collected on the Gene Hartzell Memorial Bridge was collected in a controlled manor. The research team used personal vehicles for data collection and traversed the bridge at a predetermined speed. The sensor type and placement were kept constant. Google Pixel 4 smartphones equipped with the *Good Vibrations* app were used for data collection for all trips. Each phone was secured on the dashboard with a double sided foam tape.

The controlled dataset of 332 trips across the GHMB was collected in three stages. For the first 102 trips, the vehicles traveled at an average speed of 70.56 kph with little deviation. Next, 98 trips were gathered following the speed of traffic. This speed was dependent on the level of traffic and ranged from 80 kph to 130 kph. Last, a slow speed data collection campaign was performed in collaboration with Pennsylvania Department of Transportation. Officials closed the right lane in the northbound direction to allow for data collection for speeds ranging from 16 kph to 48 kph. While collection vehicles traveled in the northbound direction, the cars drove at a prescribed speed; while traveling southbound, the vehicles traveled at the speed of traffic.

Cadore Bridge

Cadore Bridge data collection automatically occurred through daily activity of ANAS employees, and the use of the *Good Vibrations* app. In total, 884 trips have been recorded since the initiation of the campaign. The vehicle types are unknown and data collection is not controlled (e.g., speed, phone orientation, time of collection, etc.). Being a highway road, vehicle speed vary depending on traffic and activities of the ANAS employees on the bridge. The mean speed of this dataset is 62.4 kph, however, it should be noted that there are multiple trips where the mean speed is extremely low. From looking at these trips individually, the vehicles stopped on the bridge for an extended period of time. For the analysis in the main text, the samples are sorted based on their speed in ascending order and the first 200 samples are selected for the processing by the proposed method.

Ciampino Bridge

The Ciampino Bridge data collection caimpaign in the same format as the Cadore Bridge. Using the *Good Vibrations* app, 992 trips have been collected over the bridge, among which, the first 200 samples with the lowest speeds are used for the aggregation. The irregular geometry of the bridge results in vehicles driving slowly in this area and therefore, the average speed on the subset is 8.08 m/s. The data is collected in a partially-controlled fashion and no accurate information is available regarding the vehicle types.

Supplementary Note 2: Bridge Monitoring Campaigns using Fixed Sensors

In this paper, we studied four distinct bridges, with one case in reference to well-documented previous studies and three cases with no previous experimental campaigns. The Golden Gate Bridge is a landmark structure with numerous previous works dedicated to its modal identification *(1-4)*. Therefore, modal properties are adopted from one of these works and used as reference against the findings from the mobile sensing campaign. For the three new cases, comprehensive fixed sensing campaigns have been performed by the team of authors to build accurate and detailed modal references. Cadore Bridge and Ciampino Bridge in Italy have

been instrumented by ANAS S.p.A.. The instrumentation on Gene Hartzell Memorial Bridge is performed by the center for advanced technology for large structural systems (ATLSS) at Lehigh University.

Ciampino Bridge

As a part of the collaboration with ANAS S.p.A., the elevated highway intersection at Ciampino district in Rome metropolitan in Italy has been instrumented with fixed sensors. The data is used to identify natural frequencies as well as natural mode shapes. The instrumentation setup covers 30 spans of the elevated intersection, among which, two adjacent continuous spans are studied in this paper. This region is isolated via expansion joints from a neighboring span and an abutment. In total, the region is instrumented by 12 wired sensors, with six sensors equally spaced on lateral ends of the cross-section (see Figure S 2a). The fixed sensor network has been collecting triaxial vibration data since October 2020 intermittently. Successive batches of data (collected in two-hour intervals) were processed using the automated frequency domain decomposition (AFDD) algorithm *(5)* to determine the structural modal properties of the bridge and track them over time. Based on these analyses, the fundamental frequency of the bridge (see Figure S 2b) was determined at 4.5 Hz. The identified frequencies are consistent in different batches of the vibration data. This confirms that the identified values are inherent structural signatures that are independent of time. From model analysis, natural mode shapes are also identified - see Figure S 2c. The first mode at 4.5 Hz is used as reference for comparing with the mobile sensing result in Figure 3 from the main text. The amplitude of the identified modes can be detected from the power spectrum density (PSD) of the time signals. In Figure S 2d, the PSD of the first singular value of the fixed signals is plotted. The AFDD method automatically selects the prominent peaks from the PSD function of the fixed sensor signals. The selected peaks on a random batch of data are shown with red circles in Figure S 2d. The first mode at 4.5 Hz is notably stronger than other prominent peaks, and it is implied that the bridge ambient loading resonates with this frequency. This can explain the finding from the mobile sensors since the first vertical mode is one that is detected from mobile sensing data as well.

Cadore Bridge

Cadore Bridge is located in the Dolomite mountains in northern Italy. The bridge did not have an existing monitoring setup and therefore, to access ground-truth modal information, a fixed sensor monitoring campaign was conducted. In this campaign, 21 triaxial sensors are located longitudinally over the entire length of the bridge as shown in Figure S 3a. The sensors are portable and wireless MEMS devices that, based on their battery life and storage capacity, are able to continuously collect vibrations for two hours with a sampling rate of 128 Hz. The network collected vibration data for about 90 minutes. By assessing data from two primary axes (vertical and lateral), it is evident that the latter contained less noisy signal and more distinct modal peaks on the PSD (see Figure S 3d). In fact, the visual inspection of the road condition of the bridge suggests that the bridge surface is rough, which reduces the SNR in the vertical signal. In addition, by analyzing signals in the two axes multiple modal frequencies are found identical which imply 3D mode shapes with noticeable projected components in both directions. These observations are consistent with our findings from mobile sensing data - identifying one of the fundamental horizontal modes. For processing signals, the long signals from 21 channels are split into sequential snippets with a length of five minutes. Each snippet is then processed using AFDD modal identification algorithm *(5)*. The identified mode shapes and frequencies based on

the horizontal signals are presented in Figure S 3bc. For brevity, the first two mode shapes are only plotted. Note that by aggregating and processing the mobile sensing data from crossing vehicles, we are able to identify the second mode shape with 96% MAC.

Gene Hartzell Memorial Bridge

The Gene Hartzell Memorial Bridge has five spans over the Lehigh which were monitored regularly during the summer of 2021 - Figure S 4a. The data was used as a reference for identifying the natural frequencies and mode shapes. The sensor network consisted of ten wired uni-axial accelerometers secured on the bottom flange of the transverse beams along the centerline of the bridge. The sensors collected data for approximately six to eight hours per day. Similar to the analysis above the data was divided into five minute segments for analysis, and properties were tracked over time - Figure S 4b. From AFDD, the fundamental frequency was found at 0.87 Hz. The second and third modes were detected at 1.36 and 1.78 Hz, respectively. In the second phase, the sensor network was moved to the next un-instrumented spans with the goal to build complete reference for the mode shapes. Two sensors from the previous setup remained at the same location as reference sensing nodes. By combining mode shapes in a piecewise form and matching amplitudes using reference sensing nodes, full model shapes are built and shown in Figure S 4c.

Supplementary Note 3: Frequency Initialization Methods

The proposed algorithm for mode shape identification requires candidate natural frequencies (see f_k at step 7 in Table 1 of the main text) to use as reference for constructing consistent spatial patterns. These can be achieved in multiple ways: (1) by modal analysis using possibly available numerical models, (2) by referring to previous inspection reports, (3) by performing a minimal fixed sensor data collection using a few number of accessible sensing devices such as smartphones and frequency peak-picking *(6)*, or (4) by processing the crowdsourced mobile sensing data via a frequency identification method proposed in *(7)*. The last option is perhaps the most compatible solution to our presented methodology and is theoretically as applicable as the AMS identification algorithm. The other choices can result in faster and more reliable candidates, however, they are not the most flexible and scalable solutions.

In this section, the frequency identification method from *(7)* is applied on the mobile sensing data from four bridge case studies to estimate the natural frequency candidates. Note that in the original paper *(7)*, the natural frequency estimations of the Golden Gate Bridge are derived using an identical mobile sensing database. Therefore, this section covers the frequency estimation results on the remaining three bridges: Cadore Bridge, Ciampino Bridge, and Gene Hartzell Memorial Bridge. In addition, the natural frequency of the first transverse mode of the Golden Gate Bridge is identified. As shown in Figures S 2, S 3, and S 4, the fixed sensor networks are able to identify multiple modal coordinates for each bridge. However, the proposed algorithm is able to identify a subset of these coordinates using the available mobile sensing data. As a rule of thumb, modal coordinates with the highest amplitude from the PSDs in the fixed sensor signals are more likely to be discovered by the mobile sensing data.

The frequency identification results by using the most probable modal frequency (MPMF) approach *(7)* are presented in Figure S 5. Each subplot depicts the kernelized distribution of the most prominent frequencies on bridges. The references are denoted by the

blue dotted lines and the correctly identified frequencies (i.e., true positives) are depicted with red circles. In Figure S 5a, the first two most prominent peaks of the kernelized distribution match with the reference frequencies from fixed sensor data (4.70% and 2.95% error for two frequencies, respectively). In Figure S 5b, the most prominent peak is adjacent to the reference with 3.00% error. The other peaks are false positives, nevertheless, are substantially less prominent. In Figure S 5c, MPMF results for the Ciampino bridge are presented. The plot depicts a major false positive frequency identification near 3.0 Hz which is likely to be attributed to the sensing vehicle's dynamic response to the road condition on this relatively short bridge. The second identified frequency however, is a true positive detection of the first vertical modal coordinate of the bridge. This frequency is estimated with 1.68% error. Finally, in Figure S 5d, the first horizontal frequency on the Golden Gate Bridge is estimated. As expected, the frequency value is very low (0.080 Hz) and is completely disjointed from the range of vehicles dynamical frequencies. This fact enables a more reliable performance of the MPMF algorithm; the estimation error in this case is 1.87%. Note that in this case, MPMF does not identify any false positives.

References

1. AM Abdel-Ghaffar, RH Scanlan, Ambient vibration studies of golden gate bridge: I. suspended structure.J. Eng. Mech.111, 463–482 (1985).
2. SN Pakzad, GL Fenves, Statistical analysis of vibration modes of a suspension bridge using spatially dense wireless sensor network.J. structural engineering135, 863–872 (2009).
3. M Çelebi, Golden gate bridge response: A study with low-amplitude data from three earthquakes.Earthq. spectra 28,487–510 (2012).
4. E Ozer, R Purasinghe, MQ Feng, Multi-output modal identification of landmark suspension bridges with distributed smartphone data: Golden gate bridge.Struct. Control. Heal. Monit.27, e2576 (2020)
5. R Brincker, P Andersen, NJ Jacobsen, Automated frequency domain decomposition for operational modal analysis inConference proceedings: IMAC-XXIV: A conference & exposition on structural dynamics.(Society for Experimental Mechanics), (2007).
6. Y Yu, et al., Initial validation of mobile-structural health monitoring method using smartphones.Int. J. Distributed Sens. Networks 11, 274391 (2015).
7. TJ Matarazzo, et al., Crowdsourcing bridge vital signs with smartphone vehicle trips. arXiv preprint arXiv:2010.07026 (2020).

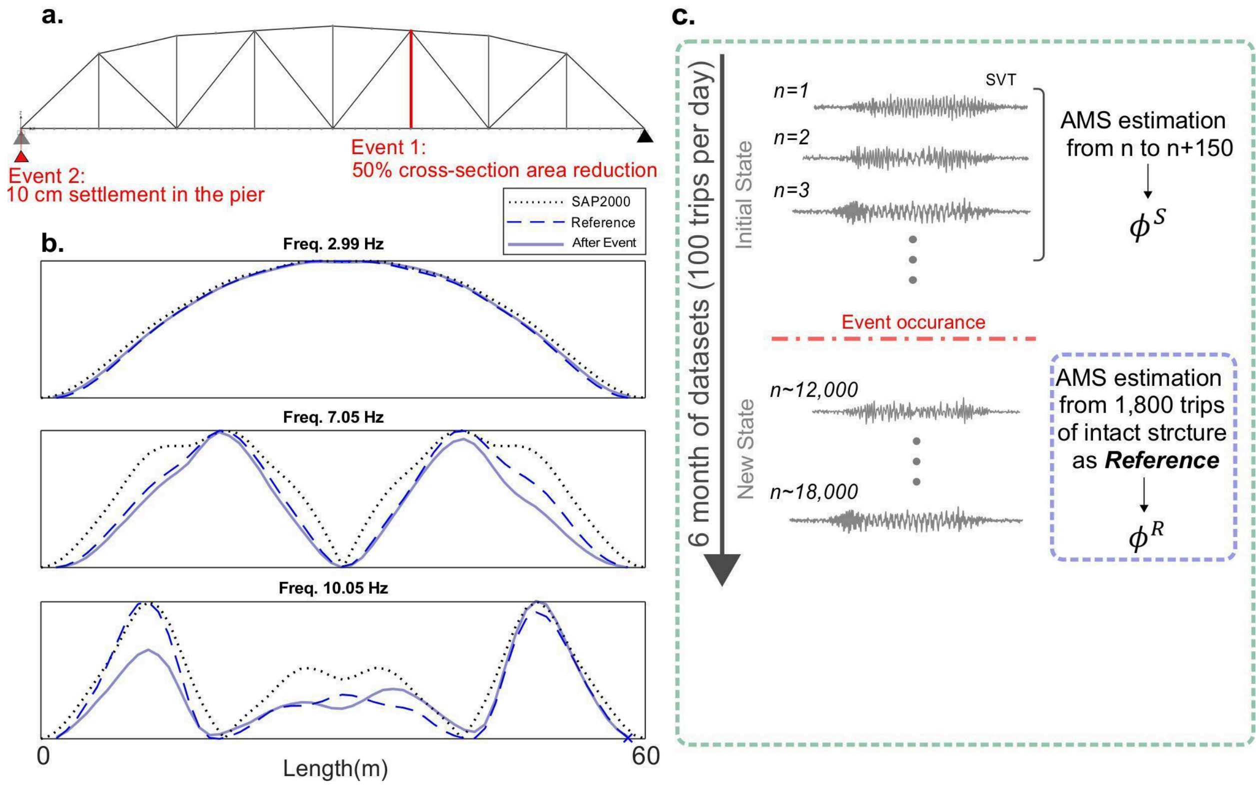

Figure S 1

Framework of numerical simulation as a potential application of the proposed method: a. Side view of the case study bridge geometry, and considered events to account for defects. b. Frequencies and absolute mode shapes of three modes. Real mode shapes (SAP2000), AMS from reference datasets, and a AMS from a sample batch after an event are also compared in each mode. c. Schematic of the procedure for anomaly detection from a large datasets that contain datasets from intact and defective structure.

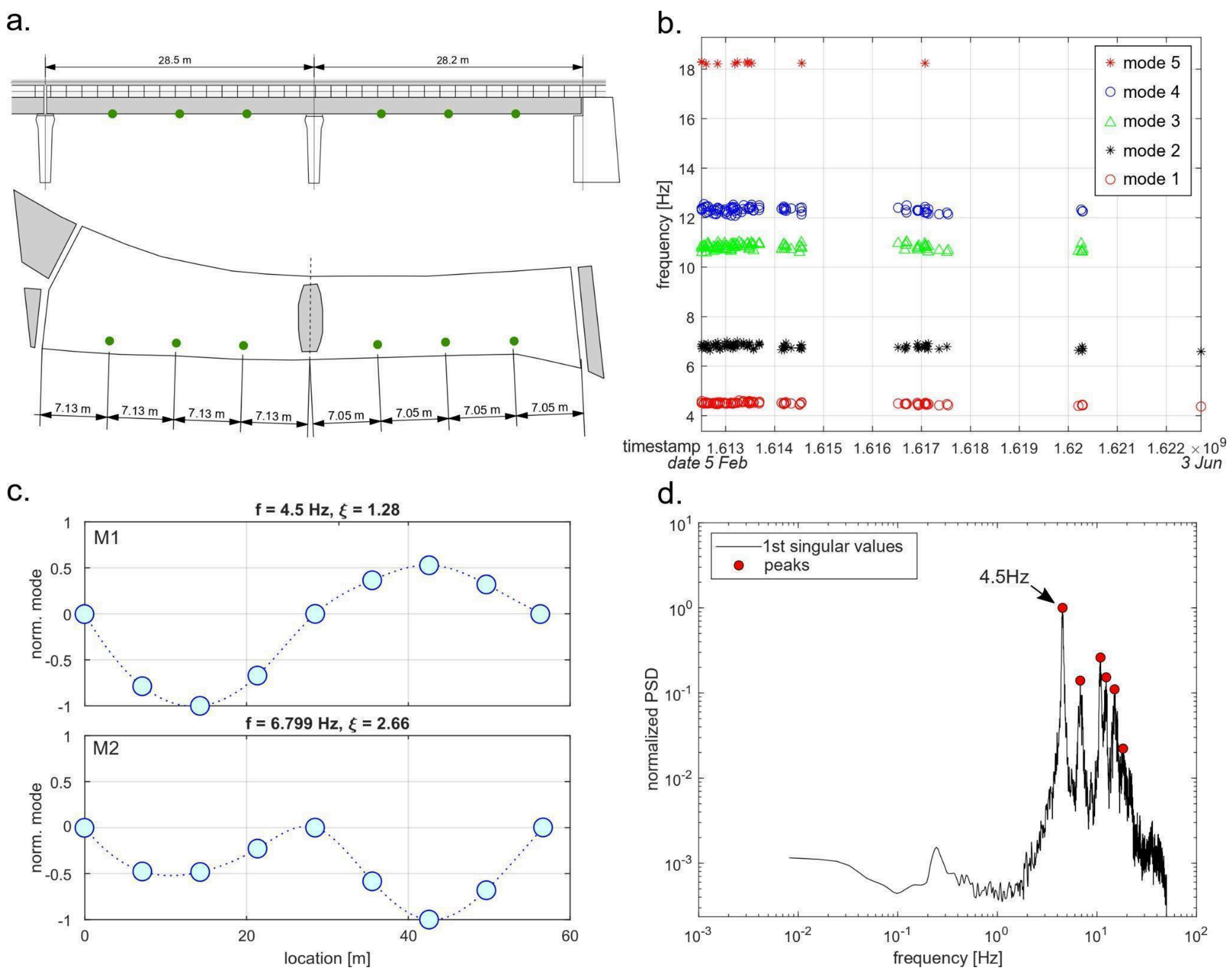

Figure S 2

Modal identification of the Ciampino Bridge using fixed sensors: a. sensors layout in plan and elevation of the Ciampino Bridge. b. timeline of the identified natural frequencies using batches of fixed sensor data in Ciampino Bridge. c. identified natural mode shapes in Ciampino Bridge. d. the spectral density function of the 1st singular value of the fixed sensor data in Ciampino bridge. The Figure shows the peaks that are selected automatically by AFDD. The first peak is strongest and is the one that is identifiable using mobile sensing data.

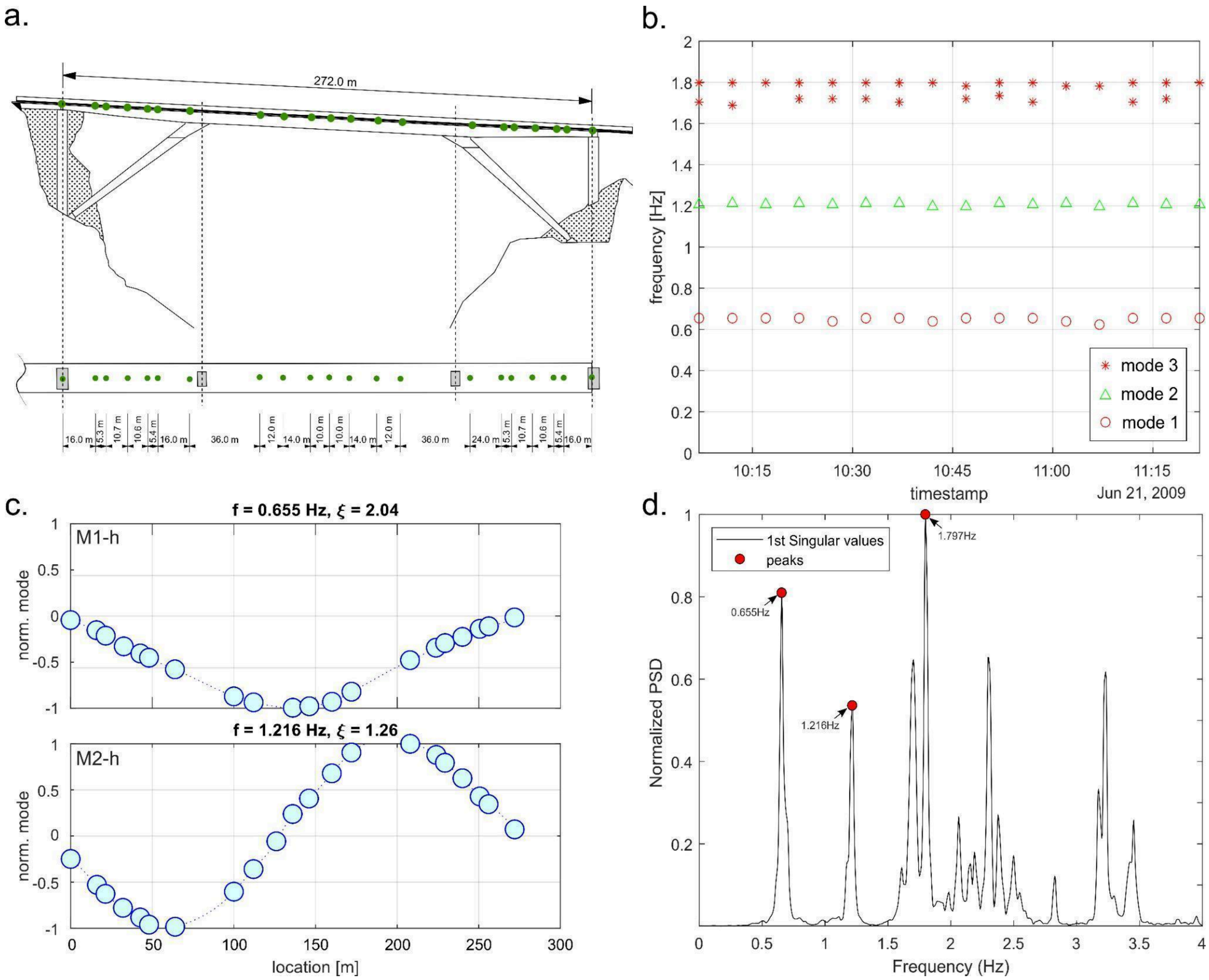

Figure S 3

Modal identification of the Cadore Bridge using fixed sensors: a. sensors layout in plan and elevation of the Cadore Bridge. b. timeline of the identified natural frequencies using batches of fixed sensor data in Cadore Bridge. c. identified natural mode shapes in Cadore Bridge. d. the spectral density function of the 1st singular value of the fixed sensor data in Cadore Bridge. The Figure shows the peaks that are selected automatically by AFDD. The second peak is one of the strongest and is the one that is identifiable using mobile sensing data.

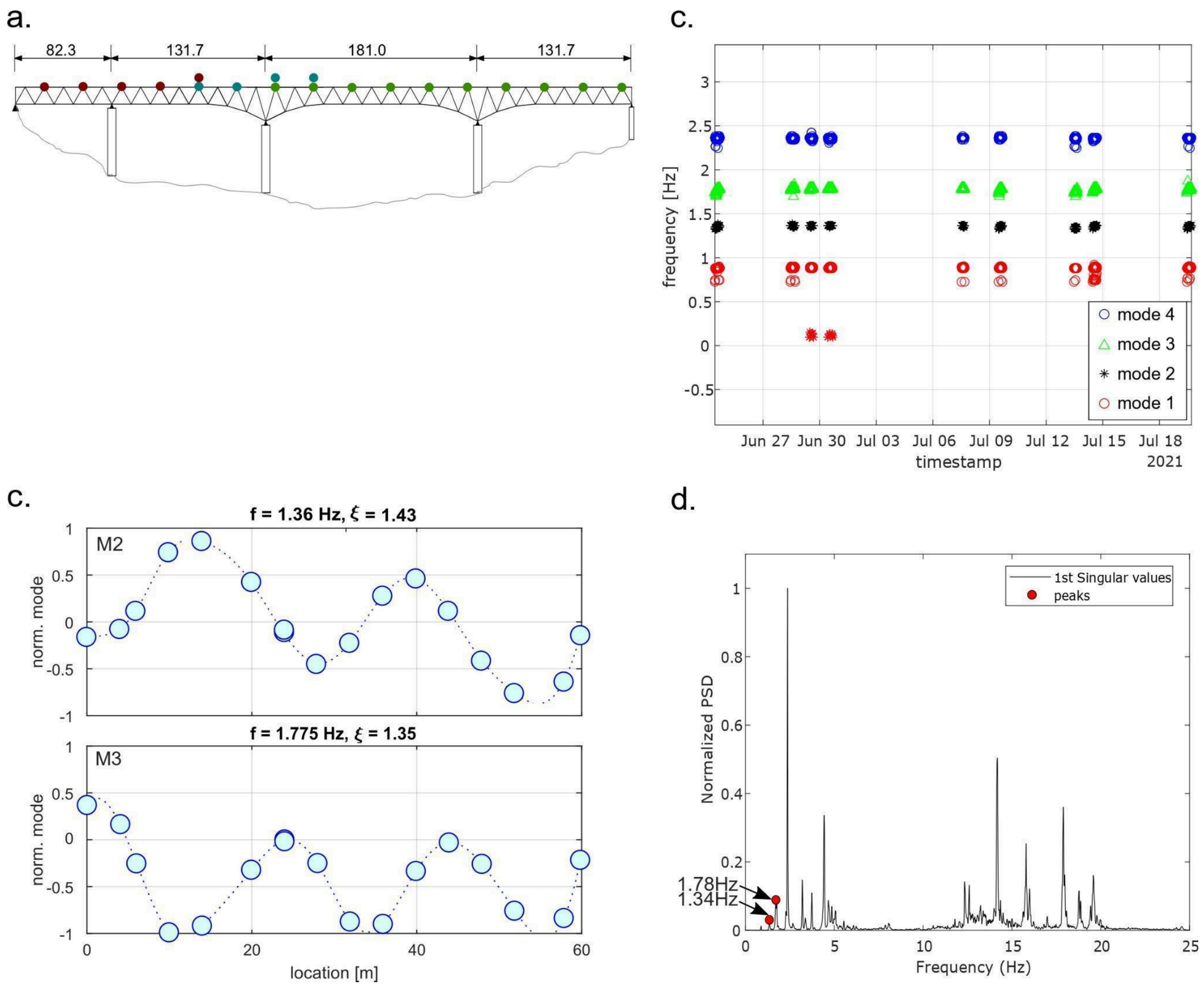

Figure S 4

Modal identification of the Gene Hartzell Memorial Bridge using fixed sensors: a. sensors layout in plan and elevation of the Ciampino Bridge. b. timeline of the identified natural frequencies using batches of fixed sensor data. c. identified natural mode shapes. d. the spectral density function of the 1st singular value of the fixed sensor data. The Figure shows the peaks that are selected automatically by AFDD. The second and third mode, at 1.34 and 1.78 Hz respectively, were identified with the mobile data.

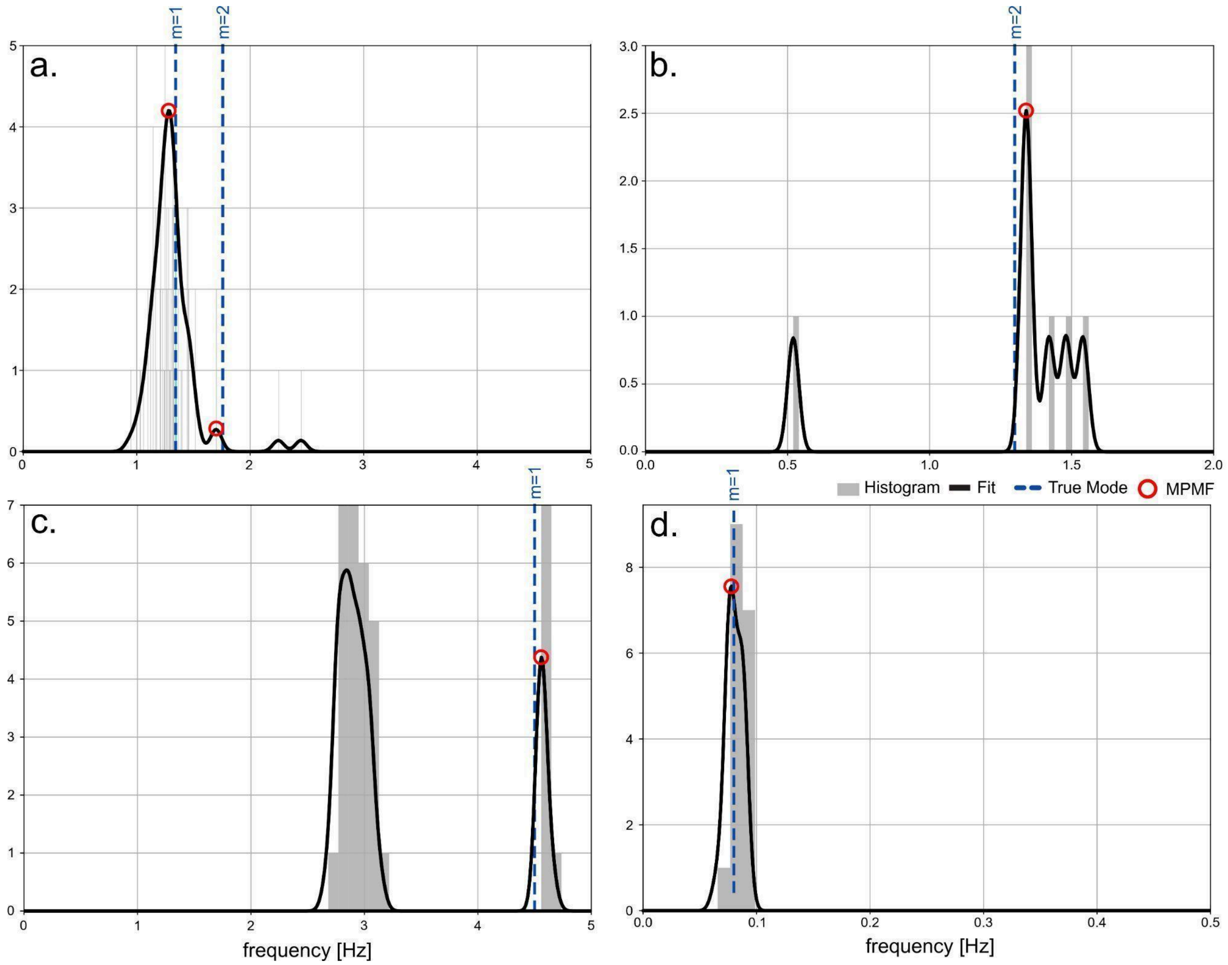

Figure S 5

Identification of the candidate natural frequencies using MPMF approach: a. Gene Hartzell Memorial Bridge: the first two natural frequencies are identified; b. Cadore Bridge: the strongest peak belongs to the second transverse frequency; c. Ciampino Bridge: the fundamental frequency is identified as one of the two most prominent peaks. It is speculated that the other prominent frequency is related to the vehicle dynamics, as suggested by *(7)*; d. Golden Gate Bridge: by aggregating acceleration signals in the horizontal direction, one prominent peak emerges that coincides with the first horizontal natural frequency according to *(1)* and *(3)*.

	f_{exp} (Hz)	f_{FE} (Hz)	Δ(%)
Mode 1	2.978	2.993	0.50
Mode 2	6.776	7.028	3.72
Mode 3	9.831	10.028	2.00
Mode 4	10.957	10.638	2.91

Table S 1

Comparison of operational modal properties measured on a bridge and the FE model used for the simulation.
